# Using Video Games to Enhance Motivation States in Online Education: Protocol for a Team-Based Digital Game

**DOI:** 10.2196/resprot.4016

**Published:** 2015-09-28

**Authors:** Anna Janssen, Tim Shaw, Peter Goodyear

**Affiliations:** ^1^ Sydney Medical School University of Sydney Sydney Australia; ^2^ Research in Implementation Science and eHealth Group Faculty of Health Sciences University of Sydney Sydney Australia; ^3^ Centre for Research on Computer Supported Learning and Cognition Faculty of Education and Social Work University of Sydney Sydney Australia

**Keywords:** digital games, medical education, online learning

## Abstract

**Background:**

Video and computer games for education have been of interest to researchers for several decades. Over the last half decade, researchers in the health sector have also begun exploring the value of this medium. However, there are still many gaps in the literature regarding the effective use of video and computer games in medical education, particularly in relation to how learners interact with the platform, and how the games can be used to enhance collaboration.

**Objective:**

The objective of the study is to evaluate a team-based digital game as an educational tool for engaging learners and supporting knowledge consolidation in postgraduate medical education.

**Methods:**

A mixed methodology will be used in order to establish efficacy and level of motivation provided by a team-based digital game. Second-year medical students will be recruited as participants to complete 3 matches of the game at spaced intervals, in 2 evenly distributed teams. Prior to playing the game, participants will complete an Internet survey to establish baseline data. After playing the game, participants will voluntarily complete a semistructured interview to establish motivation and player engagement. Additionally, metrics collected from the game platform will be analyzed to determine efficacy.

**Results:**

The research is in the preliminary stages, but thus far a total of 54 participants have been recruited into the study. Additionally, a content development group has been convened to develop appropriate content for the platform.

**Conclusions:**

Video and computer games have been demonstrated to have value for educational purposes. Significantly less research has addressed how the medium can be effectively utilized in the health sector. Preliminary data from this study would suggest there is an interest in games for learning in the medical student body. As such, it is beneficial to undertake further research into how these games teach and engage learners in order to evaluate their role in tertiary and postgraduate medical education in the future.

## Introduction

### Video and Computer Games for Educational Purposes

Over the last three decades, researchers have begun investigating how video and computer games can be utilized for nonrecreational activities such as rehabilitation, memory retention, and education [1]. The growing research interest into serious applications for digital games coincides with the rise of the “digital age,” from the mid to late 1980s onward, during which time video and computer games have evolved from existing predominantly in the domain of hobbyists into a widespread activity in contemporary society [2].

Within the field of digital game studies, a considerable amount of research has been conducted into the use of games in education [3,4]. This research has often focused on demonstrating whether games can be used for educational purposes for primary and secondary learners, with considerably less research into their use for tertiary and postgraduate education. The literature is sparser in relation to the application of digital games for continuing professional development and lifelong learning.

Current research suggests that digital games can be used effectively for educational purposes, but whether they can be tailored to teaching specific learning objectives, and the type of games best suited to doing so, remains unanswered. However, the 2014 Horizon Report [5] identified game-based learning as an area that is likely to have a major impact on learning in the next 2 years. The finding of this report is indicative of the growing interest in using digital game technology more effectively in education.

### Using Digital Games for Education Effectively

As research in this area moves forward, it is important to begin exploring what is unique about the educational experience provided by digital games compared to other educational approaches, and when and how they can be effectively utilized within a curriculum. This gap has been identified in the literature and there is a suggestion that to address it in the future, researchers need to explore the nature of the game play experience and how the player and platform interact, rather than focusing on one or the other [6].

In the area of medical education, digital games have been applied in certain formats for several decades [7]. The health sector, alongside the fields of aviation and military training, was one of the first fields to adopt a specific type of digital game for education training simulators, which began being used in health in the mid-1990s [8]. More recent research has explored the use of digital games as tools for patient education in areas such as childhood diabetes management [9]. Researchers have also demonstrated that digital games can have a positive effect for training health professionals, with a considerable interest in the medium as a tool for reducing the learning curve for surgeons [10].

There is also some suggestion in the literature that digital game-based learning may compliment the problem-based learning approach currently utilized in medical education [4]. This finding suggests there may be a role for digital games to play in postgraduate medical education in some areas. Digital games may also prove a useful tool for engaging learners in subjects that prove time intensive or contain unique concepts, terminology, or are difficult to recall. Anatomy and histology education is one such subject, with the literature indicating that retaining knowledge is considered a significant challenge for learners of the subject [11]. Additionally, anatomy content can be difficult to internalize and contain terminology learners find hard to retain [12]. Although few digital games have been utilized to teach anatomy, a nondigital card game was used to help reinforce content from lectures for optometry students learning anatomy [13]. Additionally, a study evaluated the use of a board game in a cohort of medical and dental students, and successfully demonstrated improved attitudes and perceptions toward their learning of anatomy, as well as anatomy test scores of study participants [14].

This protocol describes a study into the use of a team-based strategy game for medical education, with a particular emphasis on tertiary and postgraduate level training. The study aims to explore the player-platform and player-player interaction of an online, team-based, digital game, with the aim of identifying how digital games can be used as effective educational tools for maximum knowledge coverage. A secondary goal of the study is to evaluate the role of digital games as revision aids for adult learners and to help them assess the limits and strengths of their existing knowledge.

The use of digital games to teach adult learners is an important area for study, as the demographic has largely been overlooked in the literature thus far. In the context of adult education, there are several aspects of video and computer games that may prove valuable, but are as yet unexplored. A core element that has been researched in the field of games studies, but not significantly in educational game studies, is how players interact when playing collaborative video and computer games, a mode which has become popular in the last decade, and its impact on player engagement [15]. Educational researchers have yet to thoroughly explore these collaborative game interactions in the context of educational engagement, or in the context of existing research conducted into collaboration in education [16], but such research suggests that the collaborative and competitive elements of video games may be a core engagement element.

## Methods

### Primary and Secondary Study Outcomes

This research study uses a mixed methodology to explore the way player-player and player-platform interaction occurs in the context of educational digital games. The primary outcome of the study is to evaluate how a digital game supports player engagement, particularly its value for encouraging cooperative review of player knowledge in a specific content domain. The secondary outcome is to evaluate the impact of the game platform as a tool for adult learners to individually assess their knowledge strengths and deficits, to target revision accordingly. Additionally, the research team aims to obtain preliminary data on how digital games might be integrated into unit of study curriculums in the future.

### Game Platform of “They Know”

The game platform “They Know” is a team-based strategy game that was designed for use in a range of educational curriculums. In the context of this study, the platform will be used to develop a game for the study of anatomy and histology for medical students. The game platform distributes anatomy knowledge across a game map, using interconnected nodes that represent key learning categories. Players are divided into 2 teams and allocated to a home base at opposite sides of the map. The aim of the game is to work cooperatively with teammates in order to take control of an opposing team’s home base by crossing the map. In order to cross the map, players must answer multiple choice questions at each node they pass, related to its specific learning category.

**Figure 1:**
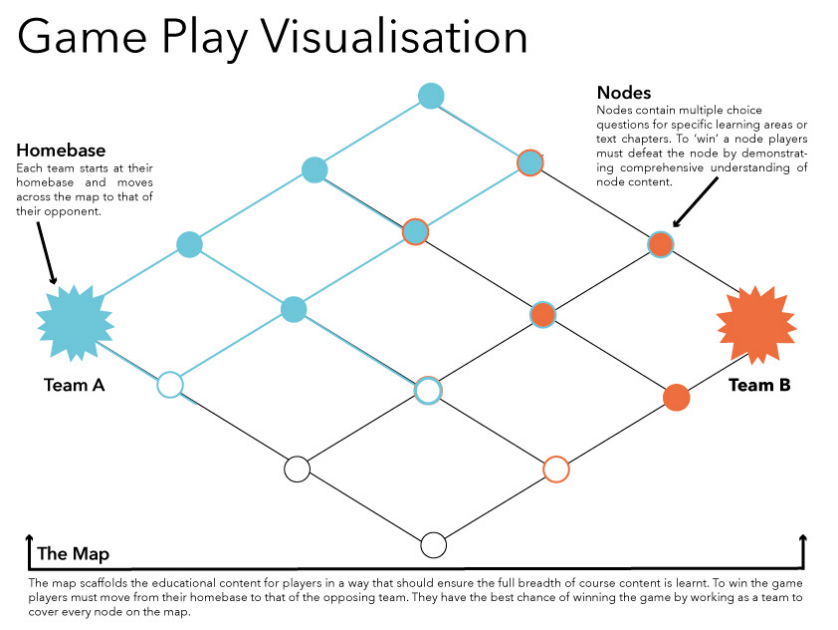
Game play diagram.

The primary population group involved in this research will be second-year medical students studying anatomy at the University of Sydney, Australia. A minimum sample size of 8 participants is required in order to complete the game session. Participation in the study is entirely voluntary (Figure 1).

In order to develop the anatomy game for the platform, an expert development group will be convened. This group will consist of subject matter experts in the area of anatomy, who are also familiar with a curriculum of relevance to the study population. The expert development group will also include educational designers who will be involved in the design of the game map and the coordination of game play sessions.

In order to obtain baseline data, participants will be asked to complete a brief Internet survey after they have consented to be involved in the study, but before they access the educational digital game. This survey will provide basic demographic information, along with data on the level of experience study participants have had with either commercial or educational video games and other online educational activities previously. Additionally, the survey will ask players to provide information on how they currently revise anatomy content, with a specific focus on collaborative approaches.

Participants will then be asked to participate in a game session. Each game session will consist of three 1-hour matches of the game, at spaced intervals. These spaced intervals will be a minimum of 2 days apart, a spacing that has been chosen to provide participants with an opportunity to do relevant revision between matches or have intermatch discussions with their teammates if they desire. Metrics collected by the game platform across the session will also be collected and analyzed to measure the impact of the game on player knowledge. Game metrics collected will include information regarding how quickly participants answered questions and how accurately.

During the game matches, players will be observed by a study coordinator and will be filmed so that the research team can review how players interact with their teammates. Filmed sessions will be analyzed retrospectively in a structured manner in order to observe incidence of player cooperation, as well as to evaluate whether this interaction was social (general chit-chat), strategic (discussion about how to navigate the map, or specific game mechanics), or educational (discussion about a players knowledge, or requests for assistance answering questions).

At the end of each match, participants will be asked to complete a Likert ranking of the match. This ranking will ask participants to rate 5 domains on a scale of 1-6: challenge, competitiveness, engagement, enjoyment, and replay likelihood. The postmatch ratings will be used to evaluate how participant engagement and enjoyment varied across the whole session.

After the conclusion of the session, participants will be asked to undertake semistructured interviews to explore their experiences with the game. The semistructured interviews will provide an opportunity to begin distinguishing how players interacted with the educational content in a unique manner as a result of using the game format. Semistructured interviews will include discussion of how cooperative game play impacted on the learning experience, as well as the value of the technology as a tool for shaping revision and review of a set curriculum. The research team will also be working with the study participants to explore the ways in which their knowledge has been structured as a result of playing the educational video game.

By conducting this study, the research team hopes to make contributions to the current understanding of video games and their use for educational purposes. In particular, it is hoped that undertaking this study will develop a more comprehensive understanding of the use of video games for educating adult learners, a group currently not significantly researched in the field of game studies. Finally, this study is researching the use of a genre of games that has never been utilized in medical education, and has only had minimal research interest in the broader educational research.

Permission to conduct this study was received from the University of Sydney’s Human Research and Ethics Committee.

## Results

This protocol describes a research study in its preliminary stages. However, several research outcomes have been achieved. First, after consultation with the medical faculty, a specific content area, first- and second-year anatomy and histology, has been chosen as the focus of the game. Additionally, an expert content development group has been convened to begin development of content for the digital game platform suitable for second-year medical anatomy students. This content group consisted of 6 subject matter experts and educational designers.

Additionally, development of the game platform has been finalized and tested by small groups of experienced commercial game players. These early tests have been used to ensure the stability of the game platform prior to being utilized by the medical students, as well as to determine expected match times for playing each game map.

Finally, second-year medical students have been recruited to participate in the study. A total of 54 second-year medical students expressed their interest in participating in the study. These individuals will be contacted to participate in small group sessions of the digital game containing the anatomy content. It is anticipated that with 54 participants it will be possible to run up to 6 sessions with 8 participants per session.

## Discussion

### Preliminary Findings

The literature on the use of educational digital games has repeatedly shown them to have a positive effect on learners [3]. In the context of medical education, there has been significant research interest in the use of digital games in clinical training, such as for surgical skills training [17]. There has also been a small amount of research into the use of digital games for continuing education, including one study into their usability for resuscitation skills retraining [18]. Far less research has been undertaken around applying digital games in the tertiary and postgraduate context, though there is some literature to suggest there is interest from students toward the use of digital games to enhance health education [19]. Although this protocol can only describe preliminary outcomes of the research study, recruitment data for the study would suggest there is some level of interest from students within the study population toward the use of a digital game to teach anatomy. This preliminary finding appears to support the existing research in this area, and has the potential to add data to an area of research that is underexplored.

At the postgraduate level, there are numerous units of study that are considered challenging for learners, but integral components of medical education. Anatomy is a good example of this phenomenon, as it is a necessary foundational subject for this student group, but learners often find it complex and difficult to learn [12]. In order to continue to offer high-quality educational experiences to learners, it is important for educators to explore tools that may enhance the educational impact of curriculums. The team-based strategy game described in this research protocol may prove a valuable tool for imparting such information, due to mechanics that make games highly engaging and the networked structure of the game map used in this genre of game, which is anticipated to align well with an anatomic curriculum.

Developing methodologies that can effectively capture how a learner interacts with a technological medium and not just the end result of that interaction is a challenge for researchers of many disciplines. In medical education digital game research, randomized controlled trials are the dominant methodology used, which are a very effective means of demonstrating a knowledge change [20]. However, they are less valuable for capturing what about the medium is unique and effective. This protocol has explored a mixed methodological approach, which may be of benefit to other health researchers interested in evaluating digital games for use on a large scale, such as in a postgraduate unit of study curriculum. The use of structured observation provided a powerful tool for developing understanding of player-player and player-platform interaction. Paired with semistructured interviews, this approach should provide a holistic perspective of how digital games can be uniquely utilized for knowledge retention and consolidation.

In all areas of digital games studies, there is a shortage of research investigating how the medium teaches effectively, particularly how various game mechanics work [21]. It is important that a better understanding of this technology’s strengths and weaknesses is developed in order to effectively utilize it in the context of medical education.

### Conclusions

Digital games are a common recreational medium in contemporary society. They have also been shown to be effective for serious applications, such as education or memory retention. In medical education, they have great potential to engage with adult learners, but there is minimal research exploring how to effectively use them for this purpose. Further research in this area would be of benefit to both learners and educators alike.
